# Improved Bat algorithm for the detection of myocardial infarction

**DOI:** 10.1186/s40064-015-1379-7

**Published:** 2015-11-03

**Authors:** Padmavathi Kora, Sri Ramakrishna Kalva

**Affiliations:** Department of ECE, GRIET, Bachupally, 500090 Hyderabad, India; Department of ECE, Velagapudi Ramakrishna Siddhartha Engineering College, Kanuru, Vijayawada, India

**Keywords:** Myocardial infarction, ECG, Improved Bat algorithm, Neural network classifier

## Abstract

The medical practitioners study the electrical activity of the human heart in order to detect heart diseases from the electrocardiogram (ECG) of the heart patients. A myocardial infarction (MI) or heart attack is a heart disease, that occurs when there is a block (blood clot) in the pathway of one or more coronary blood vessels (arteries) that supply blood to the heart muscle. The abnormalities in the heart can be identified by the changes in the ECG signal. The first step in the detection of MI is Preprocessing of ECGs which removes noise by using filters. Feature extraction is the next key process in detecting the changes in the ECG signals. This paper presents a method for extracting key features from each cardiac beat using Improved Bat algorithm. Using this algorithm best features are extracted, then these best (reduced) features are applied to the input of the neural network classifier. It has been observed that the performance of the classifier is improved with the help of the optimized features.

## Background

Heart diseases are the most important cause of human mortality globally. Every year, 9.4 million deaths are attributed to heart diseases. This includes 51 % of deaths due to strokes and 45 % of deaths due to coronary heart diseases. Most of the cardiac diseases are caused due to the risk factors such as unhealthy diet, high blood pressure, tobacco usage, obesity, diabetes and physical inactivity.

Abnormal cardiac beat identification is a crucial step in the detection of heart diseases. Normally, electrical impulses within the heart muscle stimulate the heart to contract or beat. The heart requires a constant supply of oxygen and nutrients, like any other muscle in the body. A heart attack or myocardial infarction (MI) is usually caused by a blood clot, which prevents the blood flowing through arteries or veins. Our present study describes a procedure for the detection of ECG patterns with MI. There are two types of MI in general. They are Type 1 MI (non Q type, with ST elevation and attenuated QRS complex) and Type 2 MI (Q type MI with deep Q and inverted T). This study focuses on the detection of Type 1 MI.

**Fig. 1:**
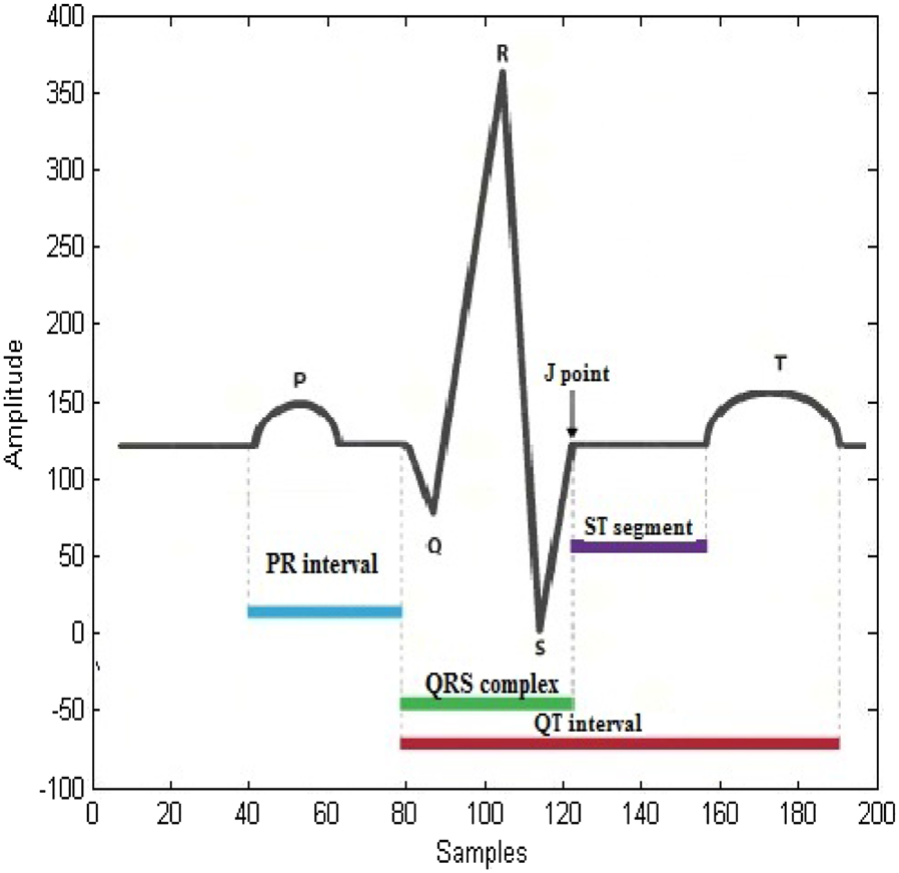
Normal ECG signal

Detection of MI using ECG involves three main steps: preprocessing, feature extraction and classification. The first step in preprocessing mainly concentrates in removing the noise from the signal using filters. The next step in the preprocessing is the segmentation of ECG file into beats and then R peaks of those segments are obtained. The samples that are extracted from each beat contains non uniform samples. The non uniform samples in each beat are converted into uniform samples of size 200 by using a technique called resampling. The resampled ECG beat is shown in Fig. 1.

Figure 1 provides information regarding amplitudes and relative time intervals of ECG. These changes in the ECG are called morphological transitions. The morphological changes (P, QRS complex, T, U waves) of ECG are due to the abnormalities in the heart. MI is one such morphological abnormality seen in the heart diseases. In the previous studies (Spilka et al. 2010) morphological features are extracted for clinical observation of heart diseases. The feature extraction using traditional techniques generally yield a large number of features, and many of these might be insignificant. Therefore, the common practice is to extract key features useful in the classification. This paper presents IBA as a feature extraction method instead of using traditional (Chatterjee et al. 2011) feature extraction techniques. In the present paper, feature extraction is based on extracting key features using nature inspired algorithm called Improved Bat algorithm (IBA) (Fister et al. 2014). Earlier studies extracted the key features using statistical and morphological techniques like Cross Wavelet transform (WT) (Banerjee and Mitra 2013), Morphological features and SVM (Spilka et al. 2010), Multiple Instance Learning (MIL) (Sun et al. 2012) which give less accuracy in the classification of MI compared to the proposed IBA.

Nature has inspired many researchers for developing algorithms to suit their needs. These algorithms are categorized basically into two types: one is based on evolutionary approach and the other is based on swarm intelligence (SI). Evolutionary algorithms mimic the Darwinian principle of “survival of the fittest” using processes of natural selection, recombination and mutation. Swarm optimization is based on the collective behavior of a group of animals. The algorithm first finds the global minimum and then searches for the best local minimum. Examples for the nature inspired optimization algorithms are ant colony optimization (Dorigo et al. 2008), particle swarm optimization (Kennedy 2010), cuckoo search (Yang and Gonzalez 2010), bacterial foraging optimization (BFO) (Passino 2002; Kora and Kalva 2015), Bat algorithm (Yang 2011), and firefly algorithm (Mishra et al. 2014; Sahu et al. 2015). IBA is a special class of swarm intelligence used for extracting a reduced feature set of 20 samples out of 200 samples taken from each beat of ECG. Without using the traditional feature extraction methods, IBA can be used for selecting the best 20 features out of 200 features in each beat. The proposed IBA can also be used for finding the most important information from a given set of samples.

There are two important things to be balanced in the nature inspired meta-heuristic algorithms (Yılmaz et al. 2014): they are exploitation and exploration. The former is used for capturing the local minimum point. On the other hand, the latter is used for finding out the global minimum. The algorithm must reach the global minimum point with out being trapped into the local minimum. A success of an algorithm depends on well balanced of these components. Too little exploration but too much exploitation may cause premature convergence, on the other hand too much exploration but too little exploitation may cause difficulties that algorithm convergences towards optimal solutions. The previous studies (Yang and Gonzalez 2010) had the advantage of exploration approach first and exploitation approach later.

**Fig. 2:**
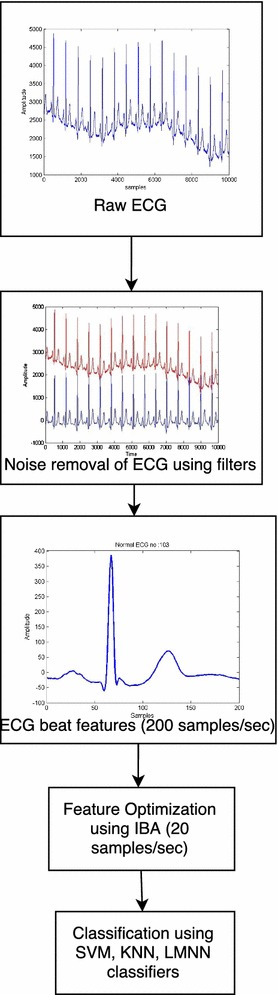
ECG classification flow diagram

Yang (2009) designed Bat Algorithm based on the behavior of micro-bats. The echo-location feature of bats is used for finding food or prey. As it approaches the prey the bat’s pulse emission rate increases and the loudness decreases. So, the pulse emission rate and the loudness of the bat can be used for selecting the optimum points in the Bat Algorithm. The aim of this study is to improve the “exploration” component of original BA so that the algorithm explores the search space more effectively. Each bat in the problem space moves with a loudness (A) and pulse emission rate (r). IBA algorithm has been designed in such way that a balance between loudness and pulse emission rate is maintained. The IBA performance can be examined by applying ECG test bench data sets and then the results are compared with BA. The ECG classification flow diagram is shown in Fig. 2.

## Methods

The proposed methodology consists of de-noising of ECG data followed by the R peak detection and beat segmentation. The heart rate is a variable quantity and accordingly the beat duration changes. So, each segmented beat is resampled into 200 samples. These beats are subjected to further analysis.

### Data acquisition

**Fig. 3:**
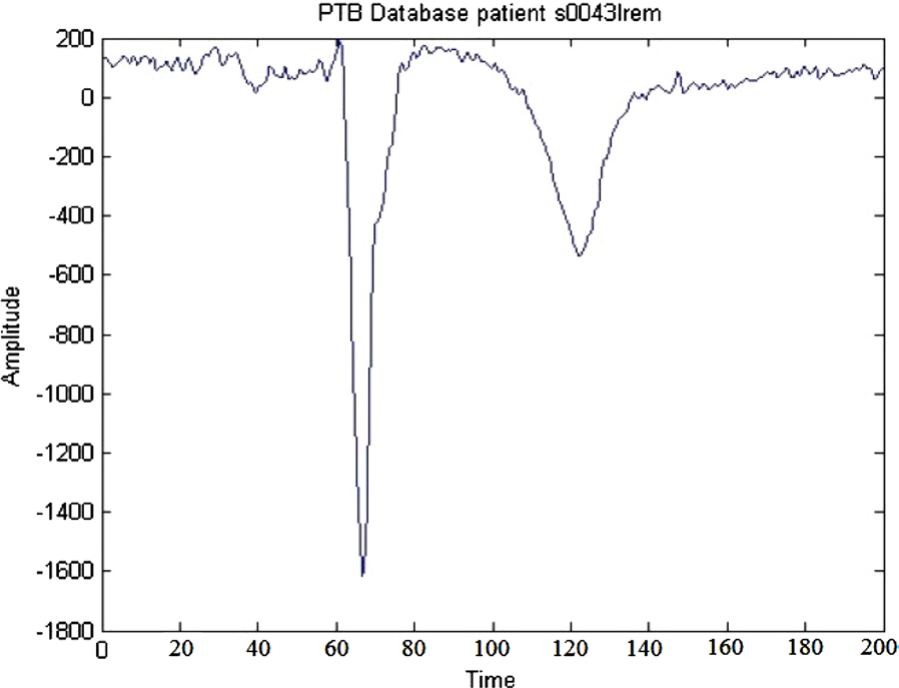
Myocardial infarction signal

The transitions in waveforms of MI ECG beats are shown in the Fig. 3.

In order to prove the performance of IBA, the usual MIT-BIH PTB database (Goldberger et al. 2000) is considered. It consists of data of 52 normal individual and that of 148 MI patients at a sampling rate of 1000 Hz. The data used in this algorithm confines to 13 recordings consisting of 6 normal and 7 MI for a duration of 30 minu. The file numbers of 13 recordings for normal and MI are shown in Table 1.

**Table 1:** MIT/BIH PTB data base

MI records	Normal records
s0043lre	s0301lre
s0088lre	s0303lre
s0100lre	s0306lre
s0235lre	s0311lre
s0242lre	s0472lre
s0386lre	s0469lre
s0559lre	

### Noise removal and R peak detection

Denoising of ECG data is a preprocessing step that removes noise and makes ECG file useful for subsequent steps in the algorithm. The Sgolay FIR smoothing filter is used for filtering.

**Fig. 4:**
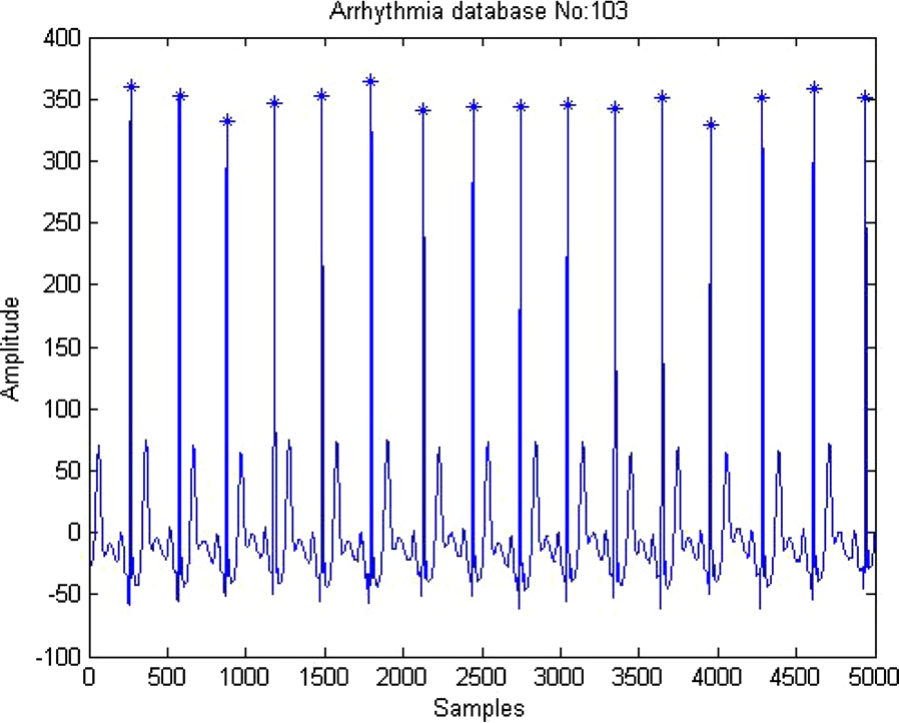
ECG R peak detection

**Fig. 5:**
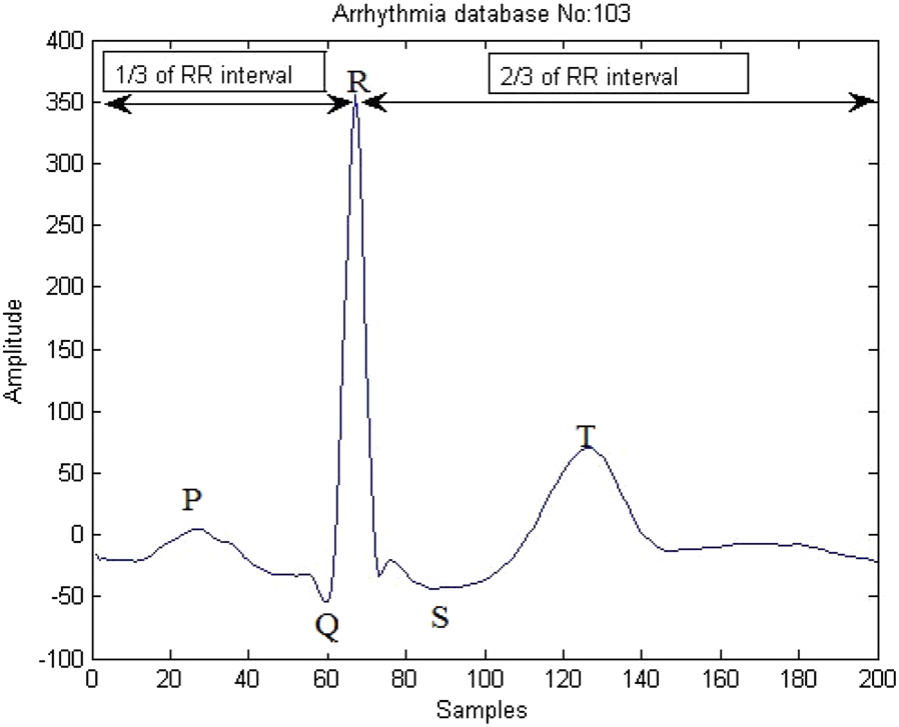
ECG beat segmentation

The R peaks of the ECG signal are detected as shown in Fig. 4. Distance between two R peaks is called RR interval. 2/3 samples to the right and 1/3 samples to the left of R peak is considered as one beat duration as given in Fig. 5.

### ECG feature selection using BAT algorithm

The customary feature extraction methods generally yields a large number of features, and many of these might be insignificant. Therefore, the effective technique in this study is to extract the key features useful in the classification of ECG beats. In this sub-section, 20 key features from 200 samples are extracted using BA, IBA algorithms.

#### Standard Bat algorithm

Bats (Yang and He 2013) are the most fascinating group of birds. They rely on echolocation. There are a total of 1200 species of bats (Fister 2013). More than half of them rely on echolocation to locate their prey.

#### Echolocation capability of bats

Most of the bats use a sophisticated sense of hearing. They release sounds that bounce(echoes) back from the insects or objects in their path. From these echoes, the bats can identify how far the insects or objects are from their current position and the also estimate the size of insects or objects within a fraction of second (Yang 2011).

#### The structure of Bat algorithm

**Fig. 6:**
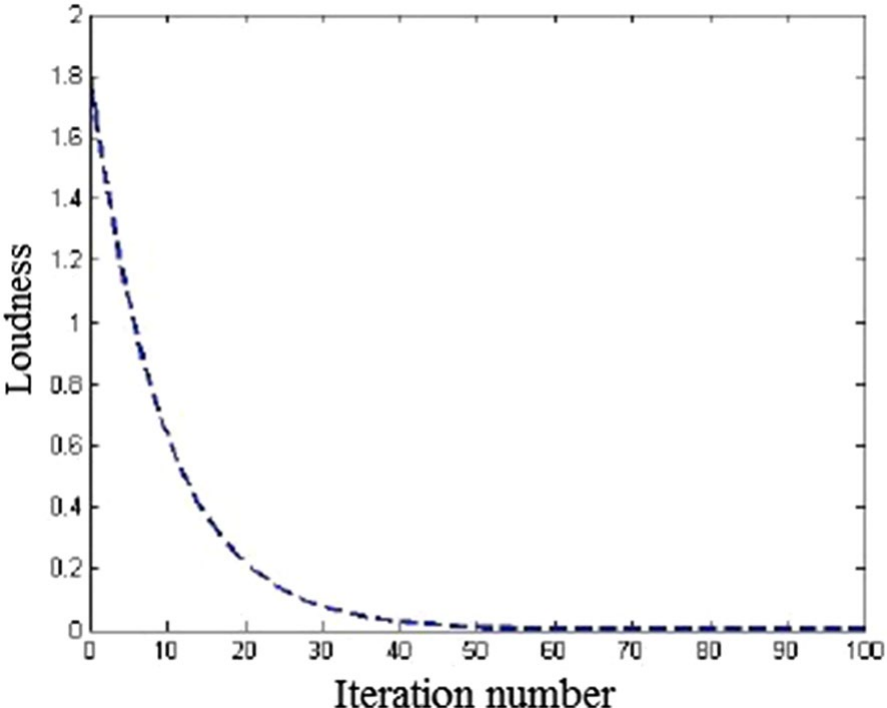
Change of loudness with iterations
- Pulse emission rate (r): The pulse emission rate $r_{i}$ has been updated using the Eq. (6) as the bats approach their food. r increases as depicted in Fig. 7. Pulse rate r yields a better solution near $X_{Gbest}$, higher pulse rate doesn’t yield best solution in local search space.
  6 $$\begin{aligned} r_{i}(t+1)= r_{i}^{0}[1 -e^{-\gamma t}] \end{aligned}$$
  where $\gamma > 0$ The r and A are updated only when the new solution is made better than the previous solution, where $\gamma$ is constant.

- Initialization: The fitness of the initial bat population is evaluated using the Rosen brock function as shown in the Eq. (1) and the values of pulse rate $r_{i}$, loudness $A_{i}$ and frequency $f_{min}$, $f_{max}$ are initialized.
  1 $$\begin{aligned} f(x)=\textstyle \sum _{i=1}^{j-1}[100(x_{i+1}-x_{i}^2)^2+(x_{i}-1)^2] \end{aligned}$$
  where j is the dimension and x(i) is the *i*th bat.
- Movement of Virtual Bats: The new bat population is generated by adjusting the position $x_{i}$ and the velocity $v_{i}$ for each bat in the population as given in Eqs. (2) and (3) respectively (lines 12–13 of algorithm 1).
  $$\begin{aligned} f_{t}= fmin + (fmax -fmin) \times rand \end{aligned}$$
  2 $$\begin{aligned} {v_{i}(t+1)= v_{i}(t) + (x_{i}(t) - x_{Gbest})f_{t}} \end{aligned}$$
  3 $$\begin{aligned} x_{i}(t+1)= x_{i}(t) + v_{i}(t) \end{aligned}$$
  The velocity and position updates of the $i$th bat are calculated using Eqs. (2) and (3). The wavelength $\lambda$ and loudness A are varied according to the location and size of the food. The $v_{i}$ and $x_{i}$ are initialized with some initial random values and a fitness function f is calculated, using the particles positional coordinates as input values. The best fitness value among all the bats is denoted as $x_{Gbest}$. The difference between $x_{i}(t)$ and $x_{Gbest}$ is a positive value which means $x_{Gbest}$ bat has more number of features than those of *i*th bat. This difference is summed up with the previous velocity to accelerate the movement of *i*th bat towards the $x_{Gbest}$bat. If the difference is negative, it means *i*th bat has more number of features than those of $x_{Gbest}$ bat. This difference is added to the previous velocity to decrease the velocity of *i*th bat.
- Local search capability of the algorithm:
  4 $$\begin{aligned} x_{new}= x_{Gbest} + \epsilon A^{-t} \end{aligned}$$
  In order to enhance the local search capability of the algorithm, Yang (2011) has created the best solution using the equation (4) where $x_{Gbest}$ is a high quality solution chosen by some mechanism. $A^{-t}$ is the average loudness value of all the bats at *t*th time step and the $\epsilon$ is generated by some random mechanism ranging between $-$ 1 and 1.
- Loudness (A): Loudness $A_{i}$ has been updated using Eq. (5) as the iterations proceed. As bats approach their food, the loudness usually decreases as depicted in Fig. 6. Generally, the loudness value will decrease when the bat starts approaching the best solution as shows in the following equation:
  5 $$\begin{aligned} A_{i}(t+1)= \alpha A_{i}(t) \end{aligned}$$
  The amount of decrease is determined by $\alpha$where $0 < \alpha < 1$.

**Fig. 7:**
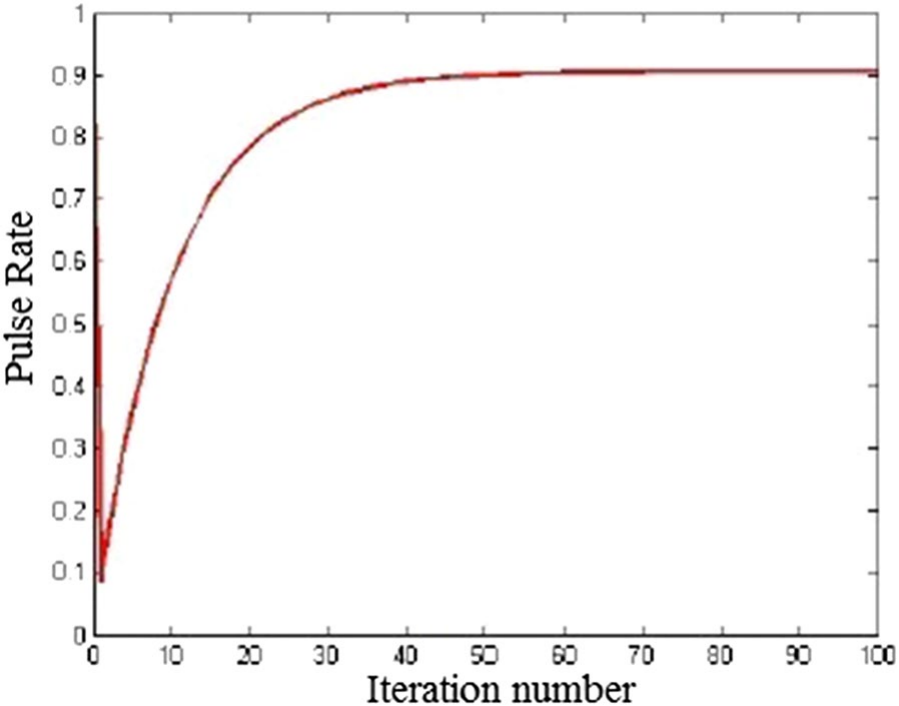
Change pulse emission rate with iterations

**Fig. 8:**
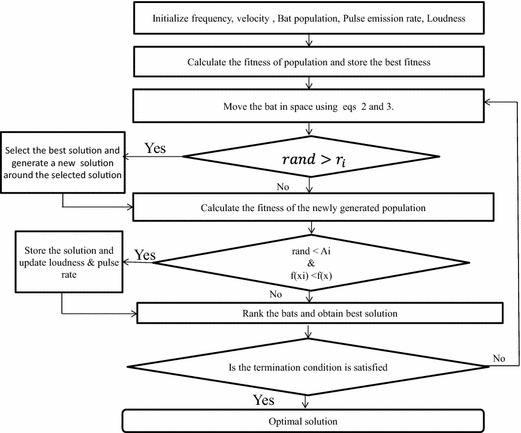
Bat algorithm flowchart

The original BA has been demonstrated in the following algorithm and the flowchart is as shown in Fig. 8. In this algorithm, the bat behavior has been analyzed based on the its fitness function. It consists of the following points:

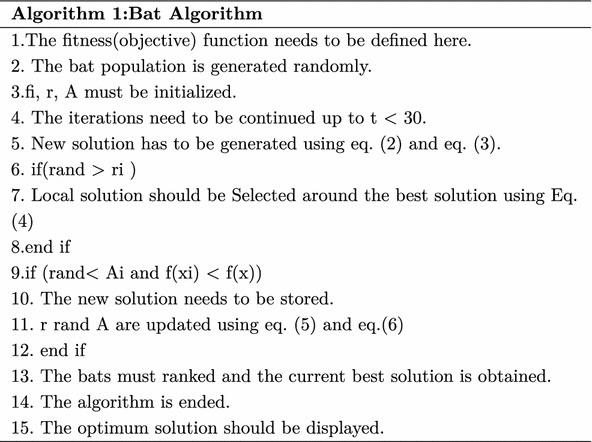



Bat Algorithm can be used for two purposes:

1. Calculating the optimum value of a function.
2. Reducing feature set of a population.

Here we are using the BA for the feature reduction.

- We have considered the initial size of Population as 2086 beats (BBB and normal).
- Load the population using Matlab command X = xlsread (‘Normal BBB. xlsx’);
- The rows of ’X’ represents the features and the columns of ‘X’ represents the size of population.
- Calculate the fitness of the above population using Eq. (1).
- The minimum value of the fitness among all the population is stored as the best value.
- Move the population to a new location using Eqs. (2) and (3).
- If rand <ri
  - Generate the local solution around the best solution using Eq. (4).
  - Else again calculate the fitness.
- if (rand <Ai) and new fitness <previous fitness
  - Store the best solution and update A and r.
  - Else rank the features of the population according to the best fitness values.

### Proposed method: improved Bat algorithm (IBA)

The global search mechanism of the algorithm is carried out by updating Eqs. (2) and (3), whereas the local search is carried out using the Eq. (4). In BA at the beginning of iterations, exploitation capability of the algorithm is high while exploration is low. It is understood that, if new candidate solutions are generated by using Eqs. (2) and (3) only, the algorithm becomes good at exploration but it turns out bad at exploitation. If new candidate solutions are generated using Eq. (4) only, then the algorithm shows good results at exploitation but it indicates bad results at exploration. Thus, the algorithm can easily get trapped into the local minimum. Actually, pulse emission rate r is a key factor which provides a balance between exploration and exploitation. It is observed that as the iterations proceed the constraint rand > $r_{i}$ decreases. Previous studies show that exploration has to be applied at the beginning and exploitation must be applied in the following iterations.

At the beginning of the iterations, A is high, so the possibility of satisfying condition rand $$< A_{i}$$ is high. At the ending of iterations, loudness A is low. So, the possibility of satisfying condition rand $$<$$$$A_{i}$$ is low. This means that the inclusion possibility of newly formed solution, which have been generated by exploration technique at the end of the iterations, into the bat population is weak. At the beginning of the iterations, if the algorithm gets trapped into the local minimum, the newly generated solution also gathers around such local minimum. Hence, the escaping possibility of BA from the local minimum decreases.

A good algorithm should have high exploration capability and high exploitation capability too in the beginning and at the end of iterations respectively. Our aim is to create an improved BA which eradicates the short comings involved in the traditional BA. Original BA is poor at exploration. Hence, the convergence towards the global optimum point is low. The exploration component of BA can be enhanced by equating pulse emission (r) and loudness (A) with the problem dimension. In BA the factors A and r, which belong to each bat, act upon all the dimensions of the solution. In the proposed approach, in order to overcome the above mentioned problem, exploration mechanism of BA has been improved by equalizing the loudness (A) and the pulse rate (r) with each dimension of the problem. The factors A and r, are assigned separately to each dimension of the population. Hence, for each dimension the exploration and exploitation operations are performed simultaneously. In IBA, local search is carried out around j dimension of the best solution. The Eq. (4), which performs local the search around the best solution in BA is updated upto dimension j as follows

7 $$\begin{aligned} x_{ij}^{t+1} = {\left\{ \begin{array}{ll} \alpha x_{ij}^{t}+\epsilon A_{j}^{-t} &{} \text {rand}_{j}>r_{ij}\\ x_{ij}^{t} &{} \text { otherwise,} \end{array}\right. } \end{aligned}$$

Eq. (7) generates an optimum value around the best solution for each dimension. where j indicates the dimension and $A_{j}^{-t}$ represents average loudness of the dimension j of solution i at time t. The processes of A and r are updated (11th line in Algorithm 1) such that they influence each dimension j of solution i. Therefore, the Eqs. (8) and (9) are updated as follows:

8 $$\begin{aligned} A_{ij}^{t+1} = {\left\{ \begin{array}{ll} \alpha A_{ij}^{t} &{} \text {rand}_{j}>r_{ij}\\ A{ij}^{t} &{} \text { otherwise,} \end{array}\right. } \end{aligned}$$

9 $$\begin{aligned} r_{ij}^{t+1} = {\left\{ \begin{array}{ll} r_{i}^{0}(1-e^{\gamma t}) &{} \text {rand}_{j}>r_{ij}\\ r_{ij}^{t} &{} \text { otherwise,} \end{array}\right. } \end{aligned}$$

### Classification of ECG features taken from the Bat algorithm

The extracted features (20 best features from 200 features) from BA, IBA algorithm are given as the input for the classifiers: SVM, KNN, LM NN, SCG NN.

#### Support vector machines

A support vector machine is a supervised machine learning method applicable for classification. It is an example for binary linear classifier, established from statistical theory of learning. It exhibits high accuracy and has capability to handle with high dimensional data sequences.

The data can be taken from II, III, and AVF leads of ECG for classifying the MI. For this study standard lead III data was taken. Total 2806 beats are used for classification. Out of total beats 1800 beats are given for training and 1006 beats are given for testing.

#### K-nearest neighbour (KNN)

In the K-nearest neighbors rule based classifier, a new vector y of a new class is classified based on the distance from nearest mean vector. The distance from vector y and the centroid of the *m*th cluster $z_{l}$ is calculated as the Euclidean distance

10 $$\begin{aligned} s_{m}=\sqrt{\sum _{l=1}^{n}(y_{l}-z_{l}^{m})^{z}} \end{aligned}$$

m is the cluster index, n is the number of the parameters used and l the parameter index. Vector y can be classified in to class k at which $s_{m}$ is minimum. We selected the value of k as 1.

#### Levenberg–Marquardt neural network (LM NN)

In this work for the detection of MI, back propagation Levenberg–Marquardt neural network (LMNN) was used. This NN provides rapid execution of the network to be trained, which is the main advantage in the neural signal processing applications (Sapna et al. 2012). The NN was designed to work well if it was built with 20 input neurons, 10 neurons in the hidden layer and 3 neurons in the output layer. The performance of this algorithm is compared with Scalar Conjugate Gradient (SCG) NN. The LM NN algorithm is a robust, and very simple method for approximating a function. SCG NN method provides conjugate directions of search instead of performing linear search. The network is trained with 1800 beats, and tested with 1006 beats. The total number of iterations was set to 1000 and Mean square error less than 0.001. The main advantage of this algorithm is that the time required to train the network is less.

## Results

### Parameter settings and analysis

The bio-inspired IBA has been implemented in Matlab 7.12.0. The experiment has been employed with a population size of 2086 for 30 generations. where increase pulse rate(r) and decrease loudness (A) have been set to 0.95. The initial value of pulse rate was set to 0.2. The $f_{max}$ and $f_{min}$ have been set to 0, 2 kHz respectively. The $A_{max}$ and $A_{min}$ value have been set to 3,1kHz respectively as shown in the Table 2.

We have selected different feature sizes for the classification. The purpose is to investigate the influence of the feature size on the classification accuracy. The accuracy was gradually decreases while the feature size increases. The accuracy value is high when features are 20 samples/beat. The original features present in each beat of ECG = [1, 2, $\ldots$, 200];

The best 20 features (column numbers) selected from IBA = [41, 14, 198, 17, 189, 139, 22, 81, 177, 117, 82, 134, 40, 49, 38, 80, 86, 129, 138].

**Fig. 9:**
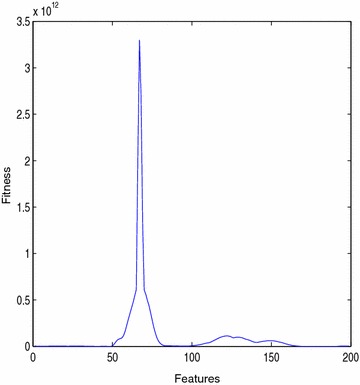
Fitness plot

The fitness values are calculated for all the bats and the maximum value of fitness of each bat is stored. The plot of fitness versus features is shown in Fig. 9. These reduced feature are given as input for the Neural Network so that its convergence speed and final accuracy can be increased.

The ECG beats after segmentation are re-sampled to 200 samples/beat. Using IBA ECG beat features are optimized to 20 features. The IBA gives optimized features (best features) for the classification. The performance of IBA is compared with classical BA technique. The BA, IBA features are classified using SVM, KNN, SCG NN, LM NN as depicted in the Tables 3, 4, 5, 6.

- Total number of beats used for classification 2806
- Count of Normal beats used for classification 1296
- Count of MI beats user for classification 1505
- Count of correctly classified beats 2754
- Total misclassified beats 52

For measuring accuracy two parameters sensitivity and specificity are calculated using the following equations.

11 $$\begin{aligned} Specificity=\frac{True\_Negative(TN)}{True\_Negative (TN)+False\_ Positive (FP)} \end{aligned}$$

12 $$\begin{aligned} Sensitivity=\frac{True\_Positive (TP)}{True\_Positive (TP)+False \_Negative (FN)} \end{aligned}$$

13 $$\begin{aligned} Accuracy=\frac{TP + TN}{TP + TN + FP + FN} \times100 \end{aligned}$$

where, TP(True_Positive) is the Count of all the correctly classified Normal beats, TN (True_Negative) is the Count of all beats the correctly classified Abnormal beats, FP (False_Positive) is the Count of Normal beats which are classified as Abnormal, FN (False_Negative) is the Count of Abnormal beats which are classified as Normal.

**Table 2:** Parameters and values

Parameter values	
Population size	2086
Generations	−30
fmin	0
fmax	1
Loudness A	−0.95
Pulse rate r	0.85

**Table 3:** Classification with KNN classifier

Classifier	Sensi (%)	Speci (%)	Accuracy (%)
BA + KNN	53.5	52.2	53.22
IBA + KNN	52.5	53.2	65.1

**Table 4:** Classification with SVM classifier

Classifier	Sensi (%)	Speci (%)	Accuracy (%)
BA + SVM	76.2	75.47	72.13
IBA + SVM	75.5	76.9	76.74

**Table 5:** Classification with SCG NN classifier

Classifier	Sensi (%)	Speci (%)	Accuracy (%)
BA + SCG NN	84.42	82.28	83.13
IBA + SCG NN	88.2	87.2	87.9

**Table 6:** Classification with LM NN classifier

Classifier	Sensi (%)	Speci (%)	Accuracy (%)
BA+LM NN	58.97	58.7	58.7
IBA+LM NN	93.342	92.2	98.9

**Fig. 10:**
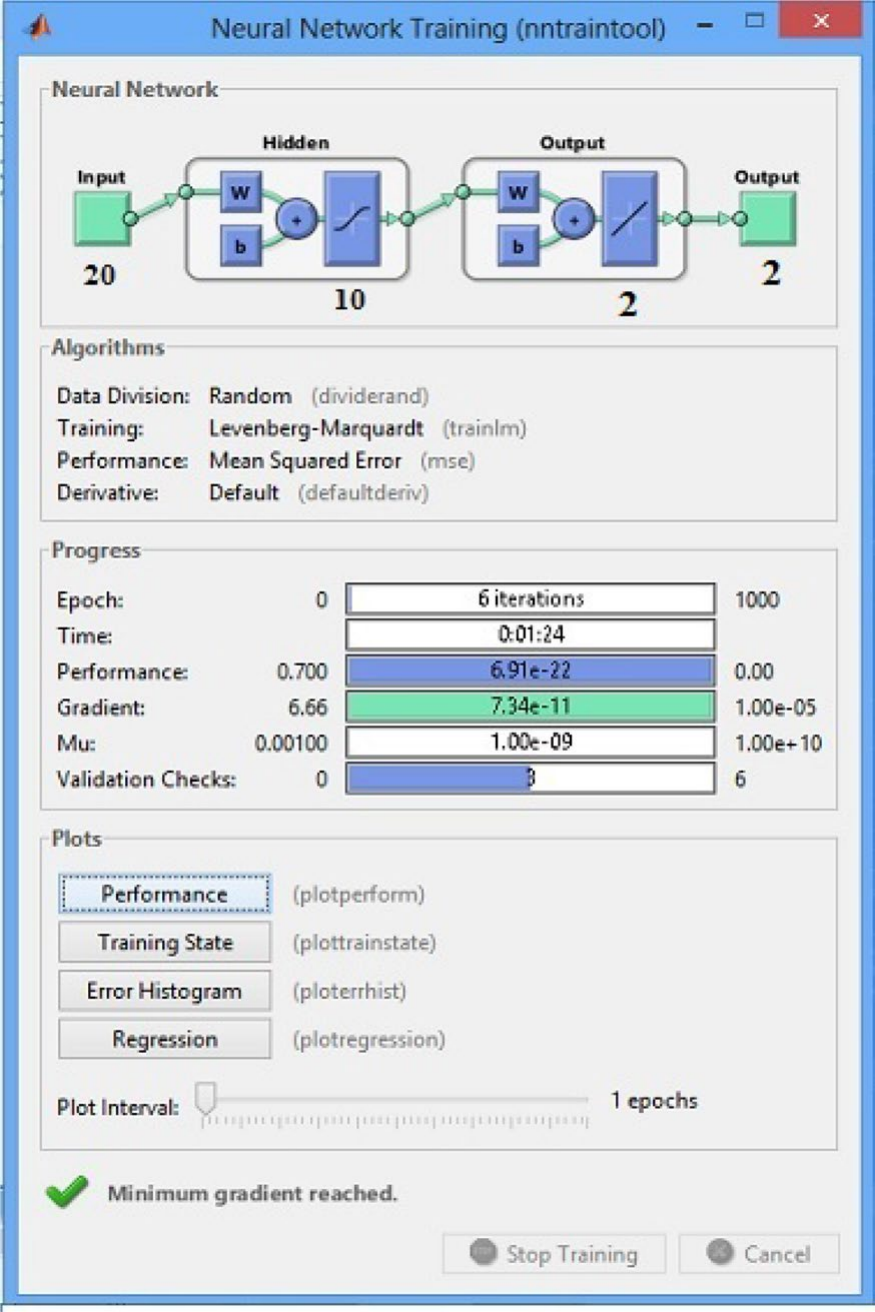
Neural network training with trainlm

In the training we applied multilayer NN, and checked the network performance and decide if any changes need to be made to the training process, or to the data sets of the network architecture. Neural network training with ’trainlm’ Matlab function is as shown in Fig. 10.

**Fig. 11:**
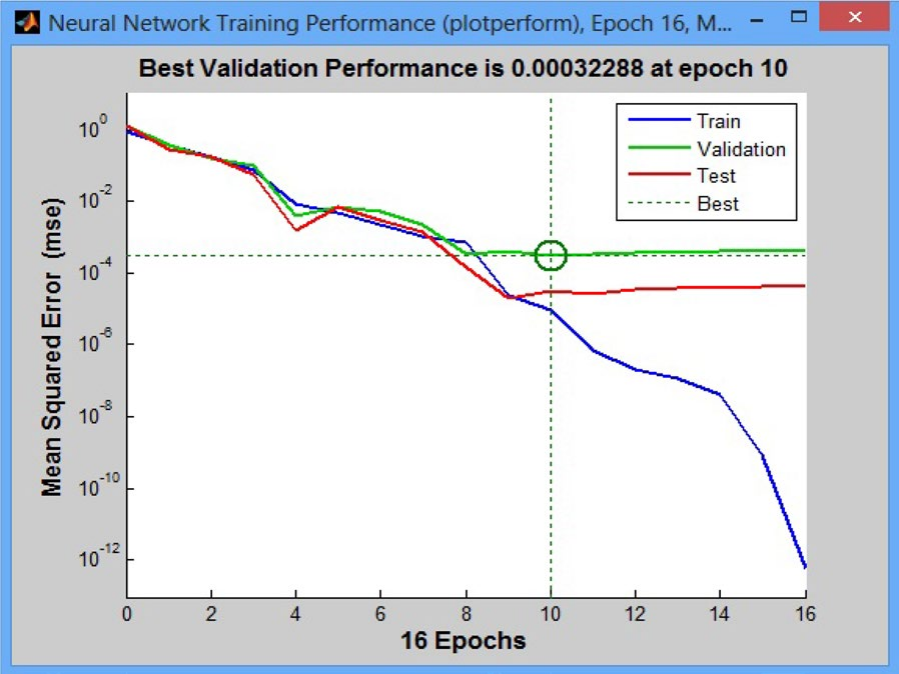
Neural network training performance plot

The property training indicates the iteration is up to the point, where the performance of the validation reached a minimum. The training is continued for 16 iterations before the stop operation. The next step is validating the network, a plot of epochs versus Mean Squared Error (MSE), which shows the relationship between the number of epochs of the network to the MSE as shown in Fig. 11. If the training is perfect, the network outputs and the targets are exactly equal but that is rare in practice.

**Fig. 12:**
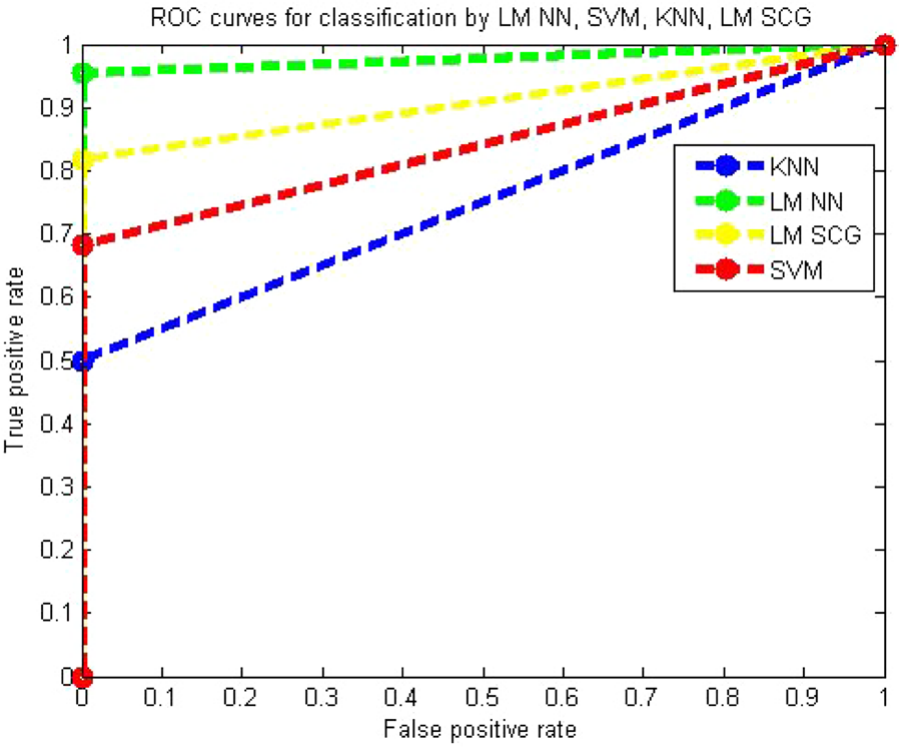
Performance comparison of different classifiers with IBA features

**Fig. 13:**
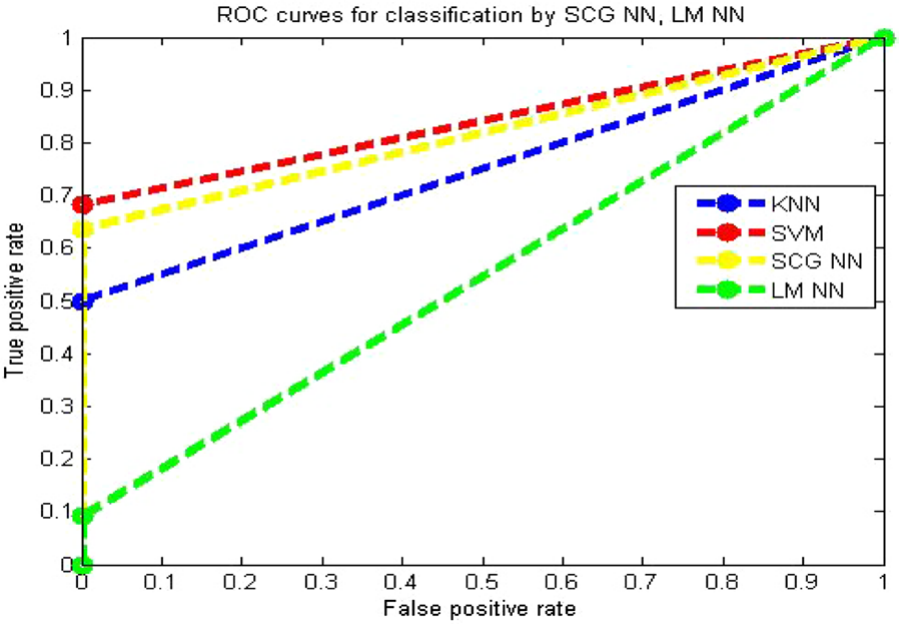
Performance comparison of different classifiers with BA features

Receiver operating characteristic (ROC) curve is plotted for true positive rate (Sensitivity) verses false positive rate (100-Specificity) as in Figs. 12 and 13. Each point on the ROC curve represents a sensitivity/specificity pair corresponding to a particular parameter. The normal and abnormal classes can be clearly distinguished using the measure of the area under the curve.

**Fig. 14:**
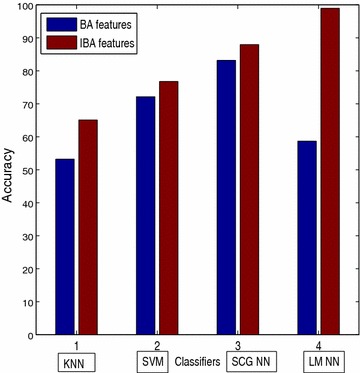
Classification accuracy bar plot

The plot of accuracy versus different classifiers shown in Fig. 14. The classification accuracy is more if IBA features are classified with LM NN classifier.

## Discussion

The proposed IBA is compared against other three MI detection algorithms as shown in the Table 7 such as Cross Wavelet Transform (XWT), Multiple Instance Learning (MIL), Morphological features and SVM in terms of related features selected from the original database and classification accuracy obtained from different classifiers using Matlab software. The work in (Banerjee and Mitra 2013), explores an experimental study of using XWT for extracting relevant features and rule based classifier for the detection of MI. The work presented in (Sun et al. 2012), uses morphological features for classification using SVM. The work proposed in (Spilka et al. 2010), uses PTB dataset taken from MIT/BIH repository and 12 morphological features are extracted then MIL is used for supervised learning and classification. From the experiments, this work concluded that the proposed IBA with LMNN classifier outperformed other three algorithms with selection of minimal number of relevant features from IBA and highest classification accuracy. The IBA employed to intelligently select the most relevant features that could increase the classification accuracy while ignoring noisy and redundant features.

**Table 7:** Comparative study for detection of MI

Studies	Approach	Accuracy (%)
Banerjee and Mitra (2013)	Cross Wavelet Transform (XWT)	87.02
Sun et al. (2012)	Multiple instance Learning (MIL)	95.86
Spilka et al. (2010)	Morphological features and SVM	86
Proposed approach	Improved BA and neural network	98.9

## Conclusion

Early changes of MI are important because immediate treatment can save the life of the patient from heart failure.There are several methods to detect features of MI. The RR interval, P wave, Statistical methods for feature extraction has some limitations.

Accurate detection of features are important for detection of MI. Nature inspired algorithms have gained increased attention of scientists and engineers in solving the problems which can not be solved by above traditional methods. In our approach IBA features for each beat(MI, normal) are extracted and the results shows that accuracy for the detection of MI has increased.

In the present study we developed a simple computational model for the detection of ECG MI using the IBA algorithm. It also projected that IBA algorithm can be used for ECG feature extraction with a view to improving its exploration behavior and decreasing the error in the final output. It can be easily observed that standard version of BA is poor at exploration, thus it is insufficient for most of the problems. In our proposed method, the modifications increases exploration capabilities at the beginning of cycles and increases exploitation capabilities towards the end of the cycles. BA and IBA are compared with bench mark ECG MI data set.

The future modifications to the algorithm are, use of multiple variants of BA such as, Gaussian distribution for random movement. The chaos enhanced BA for tuning of parameters $\alpha$, $\beta$ and $\gamma$ seems to be a better idea for solving practical problems such as feature extraction and classification.
